# Higher occurrence of nausea and vomiting after total hip arthroplasty using general versus spinal anesthesia: an observational study

**DOI:** 10.1186/s12871-016-0207-0

**Published:** 2016-07-26

**Authors:** Julien Sansonnens, Patrick Taffé, Bernard Burnand, for the ADS study group

**Affiliations:** IUMSP-Institute of Social and Preventive Medicine, Lausanne University Hospital, Route de la Corniche 10, CH-1010 Lausanne, Switzerland

**Keywords:** PONV, Total hip arthroplasty, THA, General versus spinal anesthesia, Causal effect, Propensity score, Matching

## Abstract

**Background:**

Under the assumption that postoperative nausea and vomiting (PONV) may occur after total hip arthroplasty (THA) regardless of the anesthetic technique used, it is not clear whether general (GA) or spinal (SA) anesthesia has higher causal effect on this occurrence. Conflicting results have been reported.

**Methods:**

In this observational study, we selected all elective THA interventions performed in adults between 1999 and 2008 in a Swiss orthopedic clinic under general or spinal anesthesia. To assess the effect of anesthesia type on the occurrence of PONV, we used the propensity score and matching methods, which allowed us to emulate the design and results of an RCT.

**Results:**

Among 3922 procedures, 1984 (51 %) patients underwent GA, of which 4.1 % experienced PONV, and 1938 underwent SA, of which 3.5 % experienced PONV. We found that the average treatment effect on the treated, i.e. the effect of anesthesia type for a sample of individuals that actually received spinal anesthesia compared to individuals who received GA, was ATET = 2.00 % [95 % CI, 0.78–3.19 %], which translated into an OR = 1.97 [95 % CI 1.35; 2.87].

**Conclusion:**

This suggests that the type of anesthesia is not neutral regarding PONV, general anesthesia being more strongly associated with PONV than spinal anesthesia in orthopedic surgery.

## Background

Morbidity and mortality related to anesthesia have significantly declined in recent decades [1]. While the safety and efficacy of anesthesia procedures improved, adverse events such as postoperative nausea and vomiting (PONV) have become a target to further improve quality of care. PONV may occur in 25 to 30 % of all surgery, even in 80 % for groups at risk [2]. An optimal management of PONV is important because they can lead to increased morbidity including dehydration, tension on the suture lines, hypertension, bleeding or even blindness [3]. PONV also influence negatively the quality of life of patients, for whom PONV may be harder to bear than postoperative pain [4] and cause feelings of embarrassment, humiliation or fear of subsequent interventions [5]. All these factors may induce a prolonged hospital stay, increase hospital readmission [6] and morbidity [3, 7] and thus contribute to higher health care costs [8].

Total hip arthroplasty (THA) is a frequent operation; in Switzerland, we counted 18,338 cases of hospitalization for a THA in 2012 [9]. PONV is a common adverse event in the specific context of joint arthroplasty [7]. They occur in 20 to 80 % of total joint replacements [10, 11]. THA surgery can be performed under general (GA), spinal (SA) or combined anesthesia. SA is chosen increasingly [12] because it has advantages, including a lower incidence of deep vein thrombosis and less peri- and postoperative blood loss [13]. We thus aimed to understand which factors may mitigate the occurrence of PONV after THA better. For this purpose, we used data from the ADS (Anesthésie Données Suisse) database, a large registry of routinely collected data in Swiss anesthesia departments [14].

Several studies have shown that PONV is more frequent and more severe during general anesthesia than during spinal anesthesia [15, 16], while others show no statistically significant difference [17]. General surgery patients who underwent GA were eleven times more at risk of experiencing PONV than patients who received spinal anesthesia [4]. This certainly explains why the issue of PONV is almost exclusively discussed in the context of GA, despite the increasing proportion of SA performed among all surgical procedures [18].

The purpose of this study was to assess the potential causal effect of anesthesia type (general vs. spinal anesthesia) on the occurrence of PONV in patients who had total hip arthroplasty (THA). To our knowledge, no other studies in anesthesiology have been performed in Switzerland on the issue of PONV in orthopedics with such a large number of patients.

## Methods

### Setting and participants

**Fig. 1:**
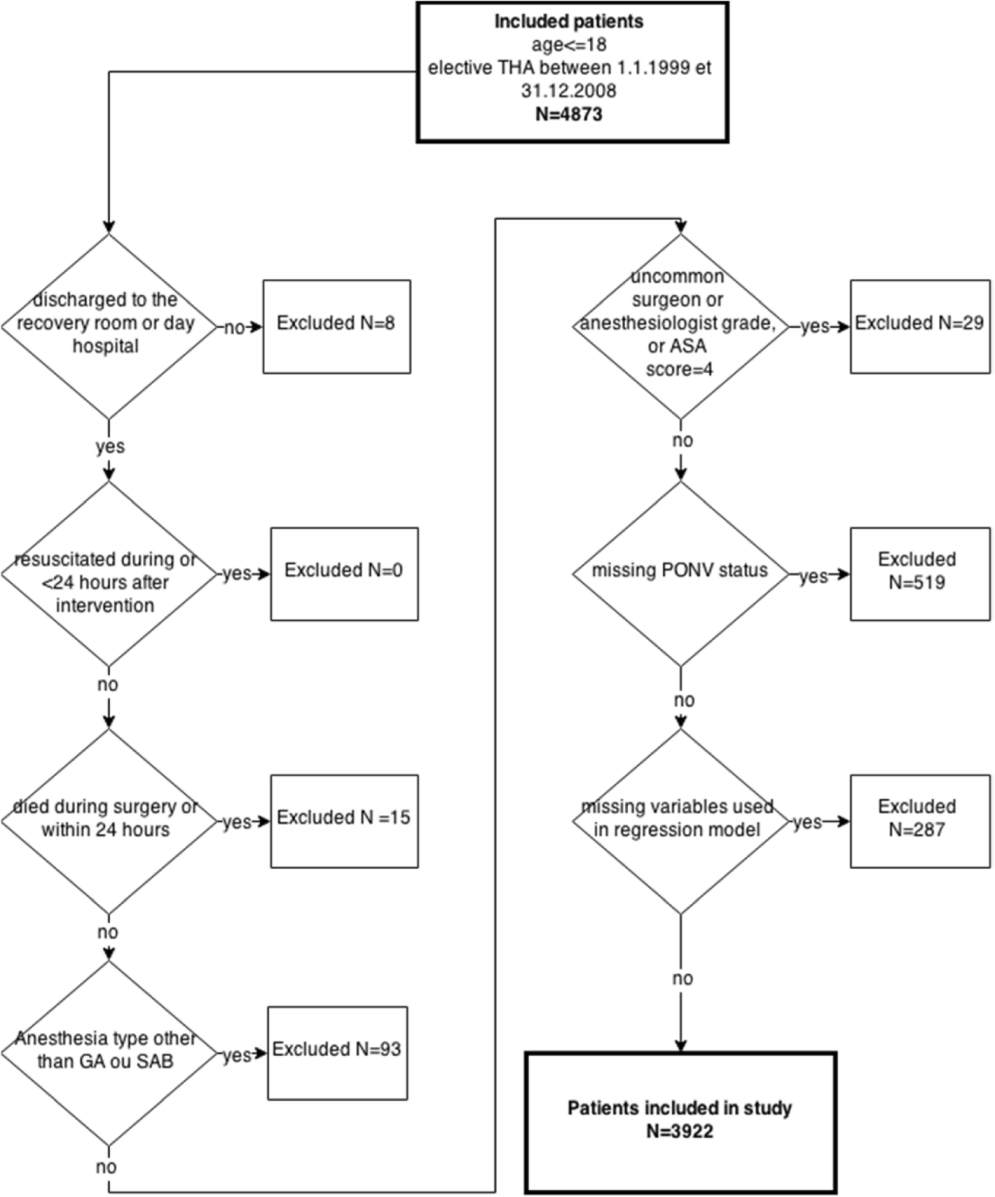
Flowchart of patients included and excluded in the study, including selection criteria

We used data collected in one orthopedic referral center specialized in orthopedics, which performs close to 20,000 interventions each year. We selected all patients above 18 years old who underwent anesthesia for elective first-intention THA (which means THA as the planned main intervention, not as a consequence or following another intervention. Bilateral THA or total hip replacement, i.e. replacing an old prosthesis by a new one, were not included) performed at any time between January 1st 1999 and December 31th 2008 (the clinic ceased participating in the ADS project from that date). Patients who have not been discharged to the recovery room, regular or intensive care ward, or day hospital, who have been resuscitated during surgery or up to 24 h later, who died during surgery or within 24 h, who underwent anesthesia type other than GA or SA (mainly combined anesthesia), with uncommon surgeon or anesthesiologist grade or ASA status 4, who lack information about PONV occurrence or other variables of interest were excluded (see Fig. 1).

### Data source

ADS was established in 1996 to foster quality improvement and for research purposes. The ADS project, supported by the Swiss Society for Anesthesia and Resuscitation (SSAR), offered its participants to share data describing various aspects of processes related to anesthesiology [14]. This database is used regularly for research purposes [14, 19, 20].

The structure of the ADS project was modular. Each participating department chose its level of participation, according to its goals and interests. For every anesthetic procedure, a standard set of core data was recorded according to common definition and format. Optionally, data related to management or quality were also collected. This system has been adapted from a similar registry system designed at the University Hospital of Trondheim, Norway [21]. Participation to ADS was open on a voluntary basis to all public and private hospitals in Switzerland. Over 40 institutions of different types and sizes participated in the project, i.e. about one fourth of all establishments practicing anesthesia in the country [22]. For the period between 1996 and 2010, more than 2 million anesthetic acts were stored in the database.

### Variables

For every procedure, the occurrence (yes or no) of an episode of PONV was routinely recorded by member(s) of the anesthesia team (nurse or physician) up to 24 h after the release of the operating theatre. However, since PONV lacks a stringent threshold-based definition, it was reported based on clinical judgment; indeed mainly clinically significant or important episodes may have been recorded. Other independent variables used were: gender, age, comorbidity indicators (allergy, angina, arterial, arrhythmia, COPD, heart disease, cachexy, diabetes, bleeding diathesis, shock, hypertension, malignant hyperthermia, infarct, infection, liver failure, renal failure, obesity, alcoholic, endocrine system, smoking, neurological disorders, steroid treatment, non-fasting, and asthma), ASA score, anesthetists’ and surgeons’ experience and calendar year. Calendar year has been included into the model to take into account temporal trends.

Data were prospectively recorded on the anesthesia chart (paper or electronic form) and stored in each department. Anonymized data were periodically transmitted securely to the data center, where they were checked for consistency and missing values. Data were then stored for further analysis [23].

### Statistical analysis

Observational studies may lead to results that differ from those of experimental studies, notably regarding the occurrence of PONV after THA performed under either SA or GA. Indeed, in an observational study, patients selected for GA generally differ from those elected to undergo SA, thus contributing to potential selection bias. Therefore, it is of utmost importance to adjust for patients’ casemix when comparing PONV occurrence rates after either GA or SA in a non randomized study. Traditionally, standard regression methods such as logistic regression analysis have been used to address this issue. However, as we shall explain in details below, standard regression methods are not well suited when the goal is to assess the causal effect [24, 25].

Traditionally, standard regression methods of the outcome on a set of potentially confounding variables have been used to assess the impact of an exposure (here GA versus SA). These methods, however, often tend to provide a biased causal estimate of the exposure as they are particularly sensitive to model misspecification, such as misspecification of the relationship between the outcome and the explanatory variables (e.g. assuming an overly simplistic linear and additive relationship, omitting important variables and interactions, etc.). In addition, they rely on strong and usually unrealistic parametric assumptions [24, 25]. The key problem that generates this model dependence is that model estimation extrapolates over regions of the variable space where observations are scarce and exposed and unexposed subjects not represented in the same way, i.e. the model extrapolates over ranges of the data that do not include both exposed and unexposed subjects and thus comparisons are very sensitive to model misspecification.

To assess the effect of GA versus SA on the occurrence of PONV, we used propensity score matching, as well as Mahalanobis distance matching (with replacement) [26, 27]. The goal of these so-called causal methods is to make the analyses in observational studies less model dependent than standard regression methods. The propensity score is the conditional probability of the exposure given a set of measured baseline covariates. Conditional on the propensity score, the distribution of the measured baseline covariates is similar between exposed and unexposed subjects [26]. Likewise, Mahalanobis distance matching allows one to balance measured baseline covariates between exposed and unexposed subjects, though somewhat more finely than propensity score matching.

The propensity score and matching methods proceed by creating a subsample of the original data in which the variables used to model the propensity score or used to perform Mahalanobis matching are equally distributed in exposed (e.g. general anesthesia) and un-exposed (e.g. spinal anesthesia) subjects, as if the exposure had been randomized. In this subsample it is, therefore, possible to compute non-parametrically the contrast (e.g. risk difference (RD) or Odds Ratio (OR)) between general versus spinal anesthesia on the occurrence of PONV. Note that balance of the unmeasured confounders will be improved in so far as they are correlated with the measured covariates included in the propensity score model.

The Mahalanobis 1-to-1 nearest-neighbor matching method proceeds directly by finding for each exposed individual one unexposed individual having the smallest possible Mahalanobis distance between the vectors of covariates, i.e. one individual whose characteristics are closest, except on the type of anesthesia received. The propensity score matching method proceeds, first, by estimating the propensity score by way of the logistic regression model, where the dependent variable is the type of exposure (1 for general and 0 for spinal anesthesia) and the regressors are the prognostic and confounding factors of the relationship between exposure and outcome (PONV, 1 yes, 0 no), then, 1-to-1 nearest-neighbor matching is performed on the propensity score and for each exposed individual an unexposed individual is selected having the smallest possible distance between the two propensity scores. Matching on the propensity score is somewhat easier than matching on the vectors of covariates, as it is a uni-dimensional problem, whereas the other is multi-dimensional. It is, however, statistically less efficient [28].

We computed the population average treatment effect (ATE), which measures the contrast (i.e. RD) of the occurrence of PONV between GA versus SA in the whole population, as well as the average treatment effect on the treated (ATET), which measures the contrast of the occurrence of PONV between GA versus SA in the subpopulation who received SA. The ATE and ATET are two measures of the effect of a treatment or an intervention commonly used in non-experimental studies [29, 30]. The latter contrast is particularly of interest to determine what would have happened had the patients who received SA instead received GA.

**Fig. 2:**
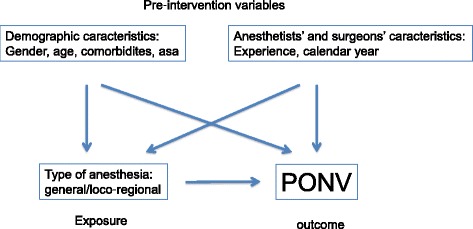
DAG of the presumed causal relationship between exposure (type of anesthesia) and outcome (PONV)

We relied on a directed acyclic graph (DAG) to select the appropriate variables to match on [31, 32], as shown on Fig. 2. The difficulty with the propensity score method lies in selecting an appropriate model so that in the matched subsample the distributions of the covariates are balanced between exposed and unexposed subjects. To achieve this goal, we included into our propensity score model all the potential confounding factors available, as well as all their two-way interactions. However, given the very large number of variables and interactions, it was not possible to reliably estimate all the regression coefficients and we selected only the interactions whose coefficients where not too large on the logit scale (e.g. not more than 3: higher values were considered as artifacts) and dropped those whose coefficient was too large (e.g. more than 3) due to the multicollinearity and curse of dimensionality [24, 25].

The final propensity score model was selected by checking that, on the one hand, the regression coefficients were not overly large and, on the other hand, that the balancing property was satisfied [33].

We computed as effect sizes the risk difference (RD) between GA and SA, and the marginal Odds Ratio (OR) comparing GA and SA. Notice that the marginal OR has not to be confused with the conditional OR, which is usually outputted by the logistic regression package. To compute the marginal OR after standard logistic regression one must marginalize [34]. The adequacy of the propensity score model was assessed graphically by comparing, after matching, on the one hand the distributions of the estimated propensity scores for those who received GA and those who received SA, and on the other hand the distributions of the covariates included into the propensity score model. All the analyses were performed using STATA software 64-bit version 13.1.

## Results

A total of 4873 procedures meeting our inclusion criteria were available in the database. After applying our exclusion criteria and discarding lines with missing values we were left with 3922 patients. Out of these patients 1984 (51 %) underwent GA, and 1938 (49 %) SA. Irrespective of the type of anesthesia, an episode of PONV was observed as a single post-anesthetic event in 93 cases, and concomitantly with one or other events in 56 cases, resulting in a total of 149 cases (3.8 %). Patients’ characteristics are summarized in Table 1.

**Table 1:** Patients’ characteristics (age, gender, ASA score, type of anesthesia and median duration of anesthesia) crossed with PONV experience status (yes/no)

Patients’ characteristics	Total (*n* = 3922)	Experienced PONV (*n* = 149)	Did not experience PONV (*n* = 3773)
Age (average ± SD)	64.2 (SD = 12.1)	63.8 (SD = 11.8)	64.2 (SD = 12.1)
Female	49.6 %	71.2 %	49.6 %
ASA score			
1	13.4 %	12.8 %	13.4 %
2	60.5 %	61.7 %	60.5 %
3	26.1 %	25.5 %	26.1 %
Type of anesthesia			
GA	50.6 %	55.0 %	50.4 %
SA	49.4 %	45.0 %	49.6 %
Median duration of anesthesia (min)	171.4	172.4	171.4

We calculated the crude odds ratio (GA vs. SA), which was OR = 1.2 95 % CI [0.87; 1.67], see Table 2.

**Table 2:** Crude odds ratio between type of anesthesia and PONV experience status

Crude odds ratio	GA	SA	Total
Experienced PONV	82	67	149
Did not experienced PONV	1902	1871	3773
Total	1984	1938	3922

**Fig. 3:**
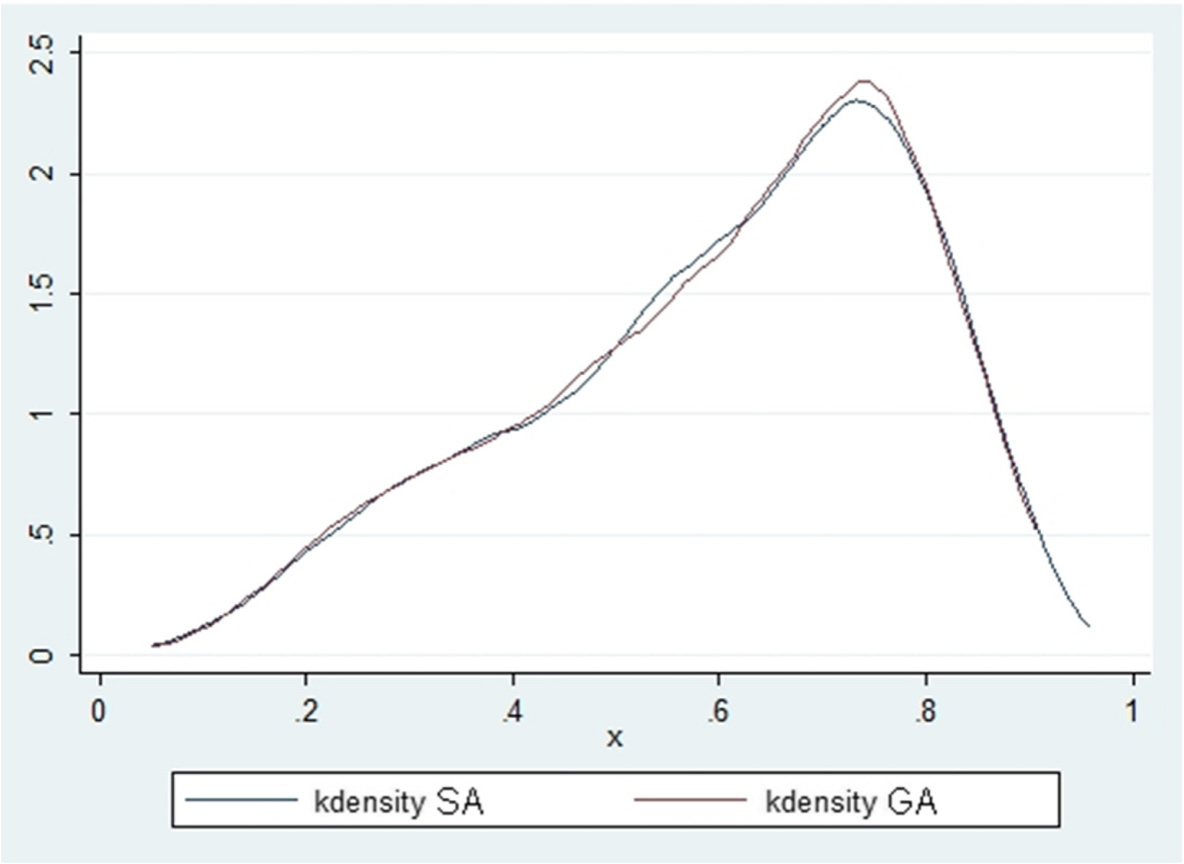
Distributions of the propensity scores after matching between SA and GA patients subgroups

After matching, the two distributions of the propensity scores were very similar in those who underwent SA (blue line) and GA (red line) (see Fig. 3).

Using the propensity score matching estimator, we found that the average treatment effect on the treated was ATET = 2.00 % with 95 % CI [0.78–3.19 %], which translates into an OR = 1.97, 95 % CI [1.35; 2.87], whereas with the Mahalanobis matching estimator we found ATET = 2.10 %, 95 % CI [0.90 %; 3.39 %], which translates into an OR = 2.10, 95 % CI [1.44; 3.12].

Regarding the average treatment effect for the 3922 patients, using the propensity score matching estimator we found ATE = 1.20 %, 95 % CI [−0.04 %; 2.49 %], which translates into an OR = 1.46, 95 % CI [1.14; 1.87], whereas with the Mahalanobis matching estimator we found ATE = 1.50 %, 95 % CI [−0.05 %; 2.96 %], which translates into an OR = 1.60, 95 % CI [1.22; 2.00]. Therefore, the risk of experiencing PONV was 1.5 ~ 2 -fold higher among patients under GA compared to patients under SA; these result show statistical and clinical significance.

Finally, with the standard logistic regression model including the same explanatory variables as in the propensity score model, as well as the interactions between them, the marginal OR was 1.12, 95 % CI [0.89; 1.41], which is considerably smaller than what we found using causal methods.

## Discussion

Relying on the registry, we found that 4.1 % of patients undergoing THA under GA and 3.5 % under SA experienced PONV. These proportions are in line with Singelyn et al. [35] who considered total hip replacement and reported PONV’s prevalences of 3, 4, and 7 % depending on the anesthetic technique used.

Our study showed a clear advantage of SA over GA for patients who underwent THA. Had those who received SA had had GA instead, the risk of PONV occurrence would have increased by about 2 %. Likewise, had everybody received GA compared to SA, the occurrence of PONV would have increased by about 1.5 %. Other studies have found contradictory results. The study from Wulf et al. [36], which compared epidural vs. general anesthesia in the perioperative management of hip replacement concluded that PONV was more common with GA, whereas Harsten et al. [37] and Fischer et al. [38] concluded the opposite. However, these studies did not use a causal methodology. Further work, ideally with RCTs, is required to clarify this issue. Nevertheless, our results clearly indicate that for patients undergoing a THA intervention under GA the incidence of PONV was higher than under SA.

Using the standard logistic regression model with the same regressors as those included into the propensity score model, it was not possible to identify the increased risk of PONV for GA. We found an OR of 1.12, 95 % CI [0.89; 1.41], which was small and non-statistically significant. This may be due to a misspecification of the functional form for the linear predictor or the omission of important confounders or interactions. The great advantages of the matching methods are that they are largely nonparametric.

Our study, however, bears several limitations. Pre-operative nausea, particularly transient nausea just after spinal anesthesia injection due to drop in blood pressure, has not been taken into account due to lack of information. Potential, uncorrelated confounders may exist that were not routinely recorded in ADS. The data have been collected over an extended period of 10 years, and it is likely that anesthetic techniques or drugs available to anesthesiologists have changed during this period. As shown by other authors, type of induction and/or maintenance agent influence the risk of PONV [39]. Using a randomized controlled trial of 1180 children and adults at high risk for PONV scheduled for elective surgery, Apfel et al. [40] concluded that in the early postoperative period, the leading risk factor for vomiting was the use of volatile anaesthetics. In the postoperative period, the use of postoperative opioids was an important predictor for vomiting. Unfortunately, these informations were not available within the ADS database. We nevertheless somehow indirectly accounted for them by adjusting for calendar year in the propensity score model. We could not determine if any of the patients in the general anesthesia group had any regional anesthesia for postop pain control, as this information was not collected in the registry. Antiemetic premedication was not collected either, and therefore the inclusion of patients who received such drugs may have introduced some bias. Demographic changes, including the aging of the population who underwent anesthesia [14], were not taken into account. It also should be noted that the quality of the collected data is likely to vary among years (depending on the turnover), although a previous study [14] showed good general quality of the ADS data. Despite a standardized definition, PONV detection relies heavily on clinical judgment, which potentially introduces an element of inter-individual and inter-hospital variability that has not yet been evaluated.

Our study also bears strengths. We used data extracted from a registry of prospectively mandatory reported pre and postoperative adverse events. Several studies have shown that the prevalence of self-reported events on a voluntary basis is generally underestimated [41–43], and such underestimation occurs irrespective of the type of anesthesia [42]. We used routinely collected data for our analyses. Studies has shown that routine data are at least as relevant to the analysis of the quality of care that clinical data collected for this purpose [44, 45], while being more cost-effective [46]. Our single-center study included 3922 acts of anesthesia, recorded over a period of 10 years, which is substantial. As a sensitivity analysis, we used two different causal methods, namely propensity score matching and Mahalanobis matching, to estimate different causal parameters (the population average treatment effect and the average treatment effect on the treated). The results were qualitatively the same and allowed us to identify a higher risk of PONV for GA versus SA; whereas this was not the case using standard logistic regression analysis. Actually, the latter is much more sensitive to misspecification of the model, such as the wrong functional form for the continuous regressors or missed interactions. Finally, unlike an RCT, our research reflects the actual daily practice of anesthesia.

## Conclusions

Using causal propensity score methods our study showed that the occurrence of PONV side effects during THA interventions was about 2 % lower for SA than for GA, a result that was not identifiable using standard regression methods only. Our results are of clinical importance given the large number of such interventions performed. Compared to other studies which included fewer patients [17], this study adds generalizability.

## Abbreviations

ADS, Anesthésie Données Suisse; ASA, American Society of Anesthesiologists; ATE, average treatment effect; ATET, average treatment effect on the treated; CI, confidence interval; COPD, chronic obstructive pulmonary disease; DAG, directed acyclic graphy; GA, general anesthesia; OD, odds-ratio; PONV, postoperative nausea and vomiting; RCT, randomized controlled trial; RD, risk difference; SA, spinal anesthesia; SSAR, Swiss Society for Anesthesia and Resuscitation; THA, total hip arthroplasty
